# Editorial: Genomics-Enabled Triticeae Improvement

**DOI:** 10.3389/fpls.2022.871816

**Published:** 2022-03-18

**Authors:** Xue-Feng Ma, Xianchun Xia, Shuyu Liu, Peter Stephen Baenziger, Hakan Özkan

**Affiliations:** ^1^Noble Research Institute, Ardmore, OK, United States; ^2^Forage Genetics International, West Salem, WI, United States; ^3^Institute of Crop Sciences, Chinese Academy of Agricultural Sciences, Beijing, China; ^4^Texas A&M AgriLife Research, Amarillo, TX, United States; ^5^Department of Agronomy and Horticulture, University of Nebraska, Lincoln, NE, United States; ^6^Department of Field Crops, Faculty of Agriculture, University of Çukurova, Adana, Turkey

**Keywords:** abiotic stress, agronomic traits, barley, biotic stress, genomics, quality traits, rye, wheat

## Introduction

Triticeae, an important tribe in the grass family Poaceae, includes several staple food crops, such as wheat, barley, rye and triticale. Although plant breeding has improved the performance of these crops steadily in agricultural traits, continued improvement is becoming more challenging due to increasing pressure from biotic and abiotic stresses as a result of climate change.

Further advance in structural and functional genomics of the Triticeae crops is essential for accelerating cycles of crop improvement and increasing genetic gain of breeding selection. Marker-assisted selection has proven to be useful for tracking alleles of major genes of many traits of agricultural importance. More recently, genomic selection has also shown potential for simplifying the selection of genome-wide minor alleles by modeling with or without pre-knowledge of the target traits. However, it has been noted that almost all Triticeae breeding programs still rely largely on conventional breeding selection made from replicated, time-consuming field trials.

With the recent advancements on reference sequences of the major Triticeae species and high-throughput genotyping platforms, we envision that more feasible genomic tools can be developed rapidly and more germplasm resources can be characterized precisely, thus, increasing the certainty of higher genetic gain through breeding. Therefore, this Research Topic aims to promote genomics-enabled Triticeae improvement by collecting original research articles, especially involving use of the most recent genomic resources in Triticeae crops.

We are honored to receive submissions of a large number of manuscripts addressing various subject areas across major Triticeae crops. After vigorous reviewing and revising, 14 of them were collected in this Research Topic, covering reports on yield components, grain quality traits, and tolerance to biotic/abiotic stresses in wheat, barley and rye.

Overall, quantitative trait loci (QTL) mapping remains a large area in this Research Topic for developing molecular breeding tools. We were also glad to receive contributions on genomic prediction modeling, especially involving multivariate prediction models covering multi-traits and multi-environments with various cross-validation schemes. These studies used different types of experimental populations such as biparental, multi-parent advanced generation inter-crosses (MAGIC) or genome-wide association study (GWAS) populations. In addition, the current Research Topic also included novel genomic resources, such as alien chromosome introgression or substitution lines. The studies characterized novel favorable alleles that are important for wheat improvement. Furthermore, a few studies reported molecular characterization of transcription factors and transporters associated with plant development and response to diverse growing conditions. These articles are highlighted below according to major traits being studied.

## Agronomic Traits

The improvement of Triticeae crops has been continuously benefited from genetic introgression of wild relatives, which provide a great potential to broaden the availability of favorable genetic alleles that are otherwise not available in the primary gene pools of the crops. In this Research Topic, Nyine et al. assessed the impact of the introgression from 21 diverse accessions of *Aegilops tauschii*, the diploid ancestor of the wheat D genome. Using whole-genome sequencing of parental lines and the sequence-based genotyping of an BC_1_F_3:5_ introgression population together with phenotyping data collected from field trials, they revealed some introgression lines that could increase grain yield. They also identified SNPs and haplotypes that were significantly associated with yield component traits and genes regulating plant development. The study provided valuable germplasm with characterized haplotypes of *Ae. tauschii* for wheat improvement.

Advancing molecular breeding tools has been a continuous task facilitating crop development. Xiong et al. located QTL associated with important agronomic traits in hexaploid wheat and developed diagnostic kompetitive allele-specific PCR (KASP) markers for the traits to facilitate wheat breeding.

In this Research Topic, agronomic traits were also mapped in rye, which is the only cross-pollinating Triticeae crop species. Siekmann et al. reported the first GWAS of agronomic traits evaluated from experimental hybrids of rye, and located cross-validated SNPs in protein-coding genes associated with plant height, heading date, grain quality and yield.

Besides QTL mapping, this Research Topic also covered genomic predication studies in wheat and barley. The winter wheat study by Gill et al. used multivariate genomic prediction models to predict several agronomic traits using advanced and elite breeding lines evaluated in multiple environments. They evaluated prediction accuracy of a multi-trait model with two cross-validation schemes and a multi-trait multi-environment model that integrates the analysis of multiple traits. Results showed that multivariate genomic selection models have great potential in implementing genomic selection in breeding programs.

In barley, genomic prediction for grain yield was modeled by Puglisi et al. using a MAGIC population derived from eight founders. Predictive abilities were evaluated for single-environment genomic prediction and multi environment genomic prediction models with various cross-validation schemes. The study concluded, in general, multi-environment models that explicitly split marker effects in main and environmental-specific effects outperform simpler multi-environment models.

## Grain Quality Traits

The Research Topic also includes studies on improving grain quality traits. Tian et al. mapped QTL for sodium dodecyl sulfate (SDS)-sedimentation volume (SSV), an important index for gluten strength of common wheat. Notably, environmentally stable QTL were detected and additive effect of two closely linked QTL on chromosome 1A was illustrated. They also characterized favorable loci for improving SSV, and proposed an ideal target for positional cloning.

In addition, Li L. et al. located QTL underneath wheat preharvest sprouting (PHS), which significantly reduces grain yield and quality. The research not only provided genetic resources for PHS resistance but also developed KASP markers tightly linked to germination index for marker-assisted breeding.

Furthermore, Halstead-Nussloch et al. revealed a novel *Gli-2* sublocus using 11 recently published chromosome-scale assemblies of hexaploid wheat. The research analyzed genomic variation in α-gliadins and unexpectedly found that the *Gli-B2* locus comprises two subloci. The research also confirmed variation of celiac disease epitopes in duplicated α-gliadin genes. The analysis yielded a new pass for improving grain quality through wheat breeding.

## Biotic Stress

Exploiting resistant resources from wild relatives has played critical roles in coping with various stresses in cereal crops. Enclosed in this Research Topic, Li J. et al. characterized wheat–*Leymus mollis* Trin. and wheat–*Psathyrostachys huashanica* Keng 3Ns (3D) substitution lines. The characterization generated new genetic resources for disease resistance and high-yield breeding with characterized substitution lines showing superior resistance to powdery mildew or *Fusarium* head blight.

Advancement in stripe rust resistance was also reported in this Research Topic. Using synthetic-derived wheats, Mahmood et al. located a large number of quantitative trait nucleotides, including some novel loci and haplotypes from *Ae. tauschii*. They also evaluated different models for genomic prediction of stripe rust resistance, and reported encouraging prediction accuracy for adult-plant resistance to stripe rust.

## Abiotic Stress

Abiotic stress is an increasing challenge in cereal production. Heat stress at booting stage causes significant losses to floret fertility (grain set) and hence yield in wheat. Erena et al. identified a major-effect heat tolerance locus on wheat chromosome 2B. The locus offsets between 44 and 65% of the losses in grain set due to heat, suggesting that it offers significant value for marker-assisted wheat breeding against heat stress.

In an effort to address wheat cold stress, Xu et al. identified genome-wide actin depolymerizing factor (ADF) genes, and characterized them using transgenic analysis. The effort generated fundamental information about the wheat ADF genes, their potential regulatory effects of the encoded proteins on plant development and responses to low-temperature stress.

In addition, Li S. et al. investigated the wheat ZIP (Zn-regulated, iron-regulated transporter-like protein) transporter, which plays an important role in regulating the uptake, transport, and accumulation of microelements in plants. The investigation searched ZIP genes against the wheat reference genome and then systematically analyzed the gene structure, expression profiles, regulatory network, and biological function regulating stress responses to microelements.

Abiotic stress was also investigated in barley. Li T. et al. studied plant mitochondrial transcription termination factor (mTERF) family, which regulates organellar gene expression. Expression analysis suggested that some members of the mTERF family were significantly induced by various abiotic stresses or phytohormone treatment, suggesting their important roles in regulating stress responses.

Altogether, the range of research in this topic clearly illustrates diverse efforts on improving Triticeae crops although the articles in this Research Topic are still very limited. The topic also highlighted the importance of public genomic resources given the fact that most of the studies involved usage of public reference genomes.

## Author Contributions

All authors listed have made a substantial, direct, and intellectual contribution to the work and approved it for publication.

## Conflict of Interest

X-FM was employed by Forage Genetics International. The remaining authors declare that the research was conducted in the absence of any commercial or financial relationships that could be construed as a potential conflict of interest.

## Publisher's Note

All claims expressed in this article are solely those of the authors and do not necessarily represent those of their affiliated organizations, or those of the publisher, the editors and the reviewers. Any product that may be evaluated in this article, or claim that may be made by its manufacturer, is not guaranteed or endorsed by the publisher.

